# Supplementary figures and images for: The Impact of Surgical Waiting Time on Oncological Outcomes in Patients with Upper Tract Urothelial Carcinoma Undergoing Radical Nephroureterectomy: A Systematic Review
Source: J Clin Med. 2022 Jul 11;11(14):4007. doi: 10.3390/jcm11144007 (PMC9323858; doi:10.3390/jcm11144007)

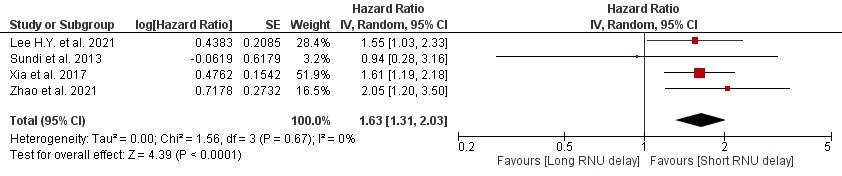

Supplement: Supplementary file 1 [file jcm-11-04007-s001.zip › jcm-1735786-supplementary.jpg]
